# Eco‐Friendly Synthesis of *Cynara scolymus* Extract–Mediated Nickel Oxide Nanoparticles for Antibacterial and Catalytic Wastewater Applications: Experimental and Molecular Docking Evidence

**DOI:** 10.1155/bca/6965791

**Published:** 2026-06-25

**Authors:** Gadah A. Al-Hamoud, Musarat Amina, Hanan M. Al-Yousef, Ayesha Mateen, Reem Hamoud Alrashoudi, Ohoud S. Alhumaidan

**Affiliations:** ^1^ Department of Pharmacognosy, College of Pharmacy, King Saud University, P.O. Box 2457, Riyadh, 11451, Saudi Arabia, ksu.edu.sa; ^2^ Department of Clinical Laboratory Sciences, College of Applied Medical Sciences, King Saud University, P.O. Box 10219, Riyadh, 11495, Saudi Arabia, ksu.edu.sa

**Keywords:** 4-nitrophenol, antibacterial, catalytic activity, *Cynara scolymus*, green synthesis, molecular docking, nickel oxide nanoparticles

## Abstract

Green synthesis of nanomaterials offers a sustainable approach for water remediation. In this study, nickel oxide nanoparticles (NiONPs) were synthesized using the *Cynara scolymus* L. head (CSH) extract via an eco‐friendly, cost‐effective route and evaluated for antibacterial and catalytic performance. Phytochemicals present in the CSH extract directed nanoparticle nucleation and growth, producing uniformly dispersed CSH‐NiONPs with enhanced functional properties. The shape and morphologies of the prepared CSH‐NiONPs were studied through different analytical techniques. A characteristic UV–vis absorption band at 328 nm was observed, while XRD analysis revealed an average crystallite size of 26.57 nm. SEM‐EDX and HR‐TEM analyses confirmed particle sizes in the range of 18–45 nm along with elemental purity. The biosynthesized CSH‐NiONPs exhibited a strong antibacterial activity against *Escherichia coli* and *Salmonella typhimurium*, with inhibition zones of 38.12 ± 0.28 mm and 41.12 ± 0.22 mm and low MICs of 9.76 and 3.12 μg/mL, respectively. Molecular docking showed that while gentamicin strongly binds aminoglycoside 3‐N‐acetyltransferase, CSH‐NiONPs interact weakly and nonspecifically, indicating minimal potential to promote resistance. Moreover, CSH‐NiONPs efficiently catalysed NaBH_4_‐assisted reduction of 4‐nitrophenol, achieving ∼95% conversion in 15 min. These findings demonstrate CSH‐NiONPs as multipurpose, sustainable nanomaterials for simultaneous microbial and chemical water remediation.

## 1. Introduction

Rapid industrialization, urbanization and technological advancement have led to the widespread contamination of aquatic ecosystems with both chemical and biological pollutants [1]. Among these, waterborne pathogenic microorganisms and toxic organic dyes represent significant threats to environmental sustainability and public health [2, 3]. The inadequate treatment of industrial effluents, agricultural runoff and domestic wastewater has further intensified the dissemination of enteric pathogens such as *Escherichia coli* and *Salmonella typhimurium* [4, 5], especially in resource‐limited regions. Although conventional disinfection methods and antibiotic therapies have historically proven effective, their efficacy is increasingly undermined by the global emergence of antimicrobial resistance and associated environmental concerns [6]. These challenges underscore the urgent need to develop sustainable, broad‐spectrum and environmentally benign antimicrobial alternatives capable of minimizing resistance development [7].

Nanotechnology offers a promising platform for addressing these limitations by exploiting the unique size‐dependent properties of nanomaterials, including high surface area, tuneable surface chemistry and enhanced reactivity [8, 9]. Metal and metal oxide nanoparticles (NPs) demonstrate strong antimicrobial and catalytic activities [10]; however, their application is often constrained by high cost, cytotoxicity and environmental persistence [11]. In this context, nickel oxide NPs (NiONPs) have gained attention due to their wide bandgap, chemical stability, mechanical robustness and broad‐spectrum antibacterial activity. Their antimicrobial mechanism is primarily attributed to the generation of reactive oxygen species (ROS), which induce oxidative stress, disrupt bacterial membranes and damage intracellular components [12]. Additionally, NiONPs exhibit remarkable catalytic efficiency in environmental remediation processes, particularly in the degradation of organic pollutants [13]. To further enhance the sustainability and biocompatibility of nanomaterials, green synthesis approaches utilizing plant extracts have been widely explored. These methods offer an eco‐friendly, cost‐effective and scalable alternative to conventional chemical and physical synthesis routes by employing phytochemicals as natural reducing, capping and stabilizing agents [14].

Herein, we report the first green synthesis of NiONPs using ethanolic extract of *Cynara scolymus* (artichoke) heads. Artichoke heads are rich in polyphenols, flavonoids, inulin and bioactive enzymes [15–18], which act synergistically as reducing and capping agents, facilitating NP formation, minimizing agglomeration and enhancing stability. This approach aligns with green chemistry principles and offers a renewable platform for producing functional nanomaterials.

Despite the inherent antimicrobial activity of plant extracts, their efficacy is generally lower than that of conventional antibiotics [19]. Gentamicin, a commonly used aminoglycoside, remains effective against Gram‐negative bacteria but is increasingly compromised by resistance mechanisms [20], particularly those mediated by aminoglycoside‐modifying enzymes such as AAC (3)‐IIa [21]. Therefore, investigating the interaction between nanomaterials and resistance pathways is crucial for developing advanced antimicrobial systems capable of overcoming drug resistance while simultaneously addressing environmental pollution.

In parallel with microbial contamination, organic dyes represent a major class of persistent aquatic pollutants due to their extensive industrial use, toxicity and resistance to biodegradation [3]. Conventional treatment methods are often inefficient and costly due to the chemical stability and complexity of these compounds [22]. Biosynthesized NiONPs have emerged as efficient multifunctional nanocatalysts, capable of accelerating dye degradation through enhanced surface reactivity and electron transfer, offering a sustainable solution for wastewater treatment [23]. The integration of biological and catalytic functionalities in green‐synthesized NiONPs highlights their potential as sustainable and efficient materials for advanced water treatment applications. This study explores the antibacterial and catalytic potential of NiONPs synthesized via the *C. scolymus* head extract (CHS‐NiONPs). While extracts from other parts of *C. scolymus* have been used for the green synthesis of silver [24, 25], gold [26] and zinc oxide [27, 28] NPs, the use of the head extract for NiONPs remains unreported. Furthermore, systematic studies on the role of *C. scolymus* head phytochemicals in reducing, stabilizing and functionalizing NiONPs, as well as their interactions with bacterial resistance enzymes, are currently lacking, representing an important research gap in sustainable nanomaterial development.

To address these gaps, this work presents a sustainable synthesis of CHS‐NiONPs, using the bioactive phytochemicals in the *C. scolymus* head extract. The NPs were thoroughly characterized using advanced spectroscopic and analytical techniques to determine their structural, morphological and physicochemical properties. Their antibacterial activity was assessed against clinically relevant pathogens and compared with both the crude extract and conventional antibiotics, highlighting the enhanced efficacy imparted by nanoscale formulation. Molecular docking was further employed to explore interactions between CHS‐NiONPs and the aminoglycoside 3‐N‐acetyltransferase enzyme (AAC(3)‐IIa), a key mediator of gentamicin resistance, providing mechanistic insights into potential interference with bacterial resistance pathways. Beyond antimicrobial activity, CHS‐NiONPs were evaluated as heterogeneous nanocatalysts for the NaBH_4_‐assisted reduction of 4‐nitrophenol (4‐NP), a model reaction for assessing catalytic performance in environmental remediation. By combining green synthesis, antimicrobial evaluation, resistance‐targeted molecular modelling and catalytic degradation of organic pollutants, this work presents a multifaceted assessment of CHS‐NiONPs. The novelty lies in the first reported use of the *C. scolymus* head extract for NiO NP fabrication, integrated antimicrobial evaluation, resistance‐mechanism targeting and catalytic applications within a single nanomaterial platform.

## 2. Materials and Methods

### 2.1. Materials and Chemicals

All chemicals, including ethanol (EtOH, ≥ 99.45%), nickel(II) nitrate hexahydrate (Ni(NO_3_)_2_·6H_2_O, 99.99%), sodium hydroxide (NaOH, 98%), 4‐NP (≥ 99%), sodium borohydride (NaBH_4_, ≥ 99.9%), potassium chloride (KCl), sodium chloride (NaCl), calcium chloride (CaCl_2_) and gentamicin (> 90%), were purchased from Sigma‐Aldrich and used as received without further purification. Fresh heads of *C. scolymus* L. (artichoke) were collected from local supermarkets in Riyadh, Saudi Arabia. Distilled water was consistently used during NP synthesis as well as for preparing 4‐NP stock solutions. The antibacterial performance of the biosynthesized CSH‐NiONPs was tested against two Gram‐negative bacterial strains, *S. typhimurium* (ATCC 14028) and *E. coli* (ATCC 25922), maintained under standard laboratory conditions.

### 2.2. Plant Material and Preparation of Ethanol Extract

Fresh heads of *C. scolymus* L. (artichoke) were procured from local supermarkets in Riyadh, Saudi Arabia, in March 2024 (imported from the Netherlands). The plant material was taxonomically identified and authenticated by a taxonomist at the Herbarium Division of College of Pharmacy, King Saud University (Riyadh, Saudi Arabia), and a voucher specimen (CS‐715) was deposited for reference. The *C. scolymus* L. heads (CSHs) were thoroughly washed, chopped, shade‐dried at room temperature and subsequently ground into a fine powder using a coffee grinder. For extraction, 50 g of dried powder was suspended in 250 mL of solvent and continuously stirred at room temperature for 24 h. The resulting mixture was filtered using Whatman No. 1 filter paper, and the extraction process was repeated three times to ensure a maximum yield [29]. The pooled filtrates were centrifuged at 6000 rpm for 15 min, and the resulting supernatant was collected and subsequently used as a biocatalyst for the synthesis of NiONPs.

### 2.3. Biosynthesis of CSH‐NiONPs

**FIGURE 1:**
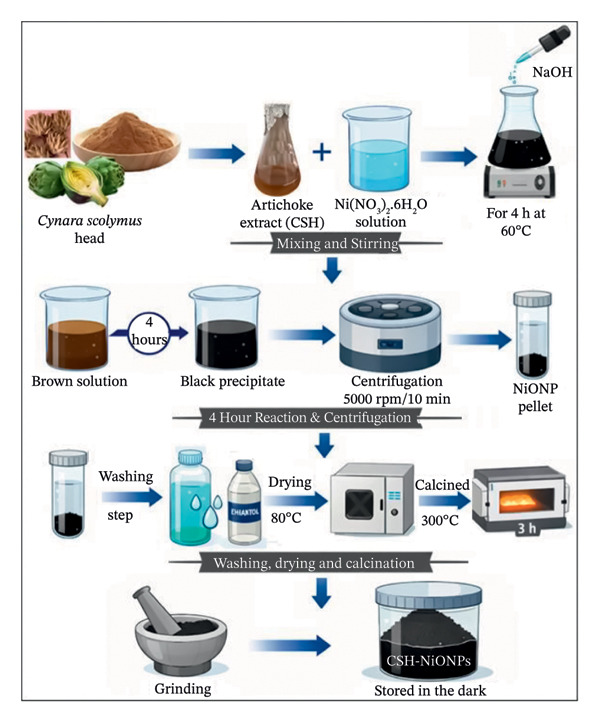
Schematic representation of the eco‐friendly synthesis of nickel oxide nanoparticles using the *C. scolymus* L. head extract as a bio‐reducing and stabilizing agent.

NiONPs were synthesized using a green‐mediated approach based on the method of Sukumaran et al., with slight modifications [30]. Briefly, 50 mL of a freshly prepared aqueous solution of nickel nitrate hexahydrate (Ni(NO_3_)_2_ 6H_2_O, 1.0 mM) was mixed with 50 mL of a freshly prepared ethanolic extract of CSHs under continuous stirring. Then, 10 mL of NaOH (1.0 M) solution was added dropwise to the mixture at 60°C with constant magnetic stirring at 500 rpm. The reaction was allowed to proceed for 4 h, during which the solution exhibited a distinct colour change from brown to dark black accompanied by precipitate formation, indicating the formation of NiONPs. The resulting CSH‐mediated NiONPs precipitate was collected by centrifugation at 5000 rpm for 10 min, and the supernatant was discarded. The obtained pellet was redispersed in distilled water and washed three times with distilled water followed by ethanol to remove residual impurities and unreacted biomolecules. The purified product was then oven‐dried at 80°C and subsequently calcined at 300°C for 3 h to improve crystallinity. After calcination, the dried material was finely ground using a mortar and pestle to obtain a homogeneous black powder. The final CSH‐NiONPs powder was stored in the dark in an airtight container in the dark for subsequent characterization and further studies. Figure 1 schematically illustrates the green synthesis of CSH‐NiONPs using an ethanolic head extract of *C. scolymus* L.

### 2.4. Characterization of CSH‐NiONPs

The biosynthesized CSH–NiONPs were comprehensively characterized using various spectroscopic and microscopic techniques. The initial formation of NPs was qualitatively evidenced by a gradual colour change in the reaction mixture. Their optical properties were analysed using UV–visible spectroscopy (UV–vis, LAMBDA‐3500 double‐beam spectrophotometer, PerkinElmer) over the wavelength range of 200–800 nm, employing a 1‐cm path‐length quartz cuvette with 1‐nm spectral resolution. Functional groups involved in the reduction, capping and stabilization of CSH‐NiONPs during green synthesis were identified via Fourier‐transform infrared (FT‐IR) spectroscopy (PerkinElmer) in the 4000–400 cm^−1^ range. The crystalline structure and average crystallite size were determined by X‐ray diffraction (XRD) using a BRUKER D8 Advance (USA) Davinci powder diffractometer with CuKα radiation (*ʎ* = 0.15406 Å) over a 2*θ* range of 20°–80°. The average crystallite size (*D*) was calculated using the Debye–Scherrer equation, *D* = *Kʎ*/*β* cos*θ*, where *D* represents the crystallite size, *K* = (0.9), *ʎ* is the X‐ray wavelength (1.5406 Å), *β* is the full width at half maximum (FWHM) of the diffraction peak, and *θ* is the Bragg angle. The surface morphology and elemental composition were examined by scanning electron microscopy (SEM) coupled with energy‐dispersive X‐ray spectroscopy (EDX) on a LYRA3 TESCAN system at 15 kV. The detailed internal structure, size distribution and morphology were further investigated by transmission electron microscopy (TEM, JEOL‐1010, Japan). For TEM analysis, a drop of the CSH‐NiONP suspension was placed on a carbon‐coated copper grid and air‐dried before imaging.

### 2.5. Antibacterial Activity

The antibacterial activity of the green‐synthesized CSH‐NiONPs was assessed against two Gram‐negative bacterial strains, *S. typhimurium* (ATCC 14028) and *E. coli* (ATCC 25922), using the agar well diffusion assay according to the protocol of Hossain et al. [31]. The bacterial strains were obtained from the Department of Clinical Laboratory Sciences, King Saud University. Each strain was subcultured on nutrient agar and incubated at 37°C for 24 h. After incubation, a single well‐isolated colony from each culture was aseptically transferred and uniformly spread onto Mueller–Hinton agar (MHA) plates (Oxoid, UK) using a sterile swab to achieve a standardized inoculum of 1.5 × 10^8^ CFU/mL (0.5 McFarland standard). After that, wells of 8 mm diameter were aseptically punched into the agar using a sterile cork borer, and 100 μL of CSH‐NiONP stock suspension (10 mg/mL) was added to each well. The plates were then incubated at 37°C for 24 h, with Gentamicin (10 μg/mL) serving as a positive control. Antibacterial efficacy was determined by measuring the diameter of the clear zones of inhibition (mm) surrounding each well.

#### 2.5.1. Determination of Minimum Inhibitory Concentration (MIC) and Minimum Bactericidal Concentration (MBC) of CSH‐NiONPs

The MIC of CSH‐NiONPs was determined using the broth microdilution method in sterile 96‐well polystyrene microtiter plates against *S. typhimurium* and *E. coli*. A stock suspension of CSH‐NiONPs (10 μg/mL) was prepared in Mueller–Hinton broth (MHB), which served as the growth medium. Wells 2–12 were filled with 100 μL of MHB, while Well 1 contained 200 μL of the NP suspension. A twofold serial dilution was performed from wells 1 to 10 by transferring 100 μL sequentially between adjacent wells. Thereafter, 10 μL of standardized bacterial inoculum was poured to all wells except well 12. Well 11 served as the positive control (MHB with bacterial inoculum), while Well 12 served as the negative control (MHB only). The plates were incubated at 37°C for 18–24 h. Following incubation, 40 μL of freshly prepared MTT solution (0.5 mg/mL in sterile distilled water) was added to each well and further incubated at 37°C for 30 min. Bacterial growth was assessed based on the reduction of the yellow tetrazolium salt (MTT) to purple formazan crystals by metabolically active cells. The MIC was defined as the lowest concentration of CSH‐NiONPs that completely inhibited visible bacterial growth, as indicated by the absence of formazan formation [32, 33]. Following the determination of the MIC, a 30‐μL aliquot from each MIC well showing no visible bacterial growth was aseptically subcultured onto MHA plates and incubated at 37°C for 24 h. In parallel, bacterial cultures treated with CSH‐NiONPs at the MIC level, along with untreated controls, were also plated on MHA plates. The MBC was defined as the lowest concentration of CSH‐NiONPs, resulting in ≥ 99.9% bacterial cell death. MBC determination was achieved by assessing bacterial survival through the visual inspection of colony formation on agar plates before and after incubation.

### 2.6. Molecular Docking

The three‐dimensional crystal structure of the target receptor, AAC(3) (PDB ID: A1YKW2), was retrieved from the UniProt Knowledgebase (UniProtKB) in Protein Data Bank (PDB) format (UniProt, 2006). The chemical structures of the ligands, gentamycin (ChemSpider ID: 65328) and CSH‐NiONPs (ChemSpider ID: 14121), were obtained from the ChemSpider database in MOL file format (‘Gentamicin C1’, n.d.; ‘Nickel(II) oxide’, n.d.). The ligand structures were converted from MOL to PDB format using Open Babel Version 2.4.1 [34] and subsequently subjected to energy minimization using Avogadro software to achieve geometrically optimized conformations [35, 36]. Prior to molecular docking, receptor preparation was carried out using AutoDock Tools v1.5.7 [37], wherein all crystallographic water molecules, heteroatoms and cocrystallized ligands were removed to eliminate potential interference during docking. Polar hydrogen atoms were added, and Kollman partial charges were assigned to the receptor. The prepared receptor structure was then saved in PDBQT format. Ligand preparation involved the addition of Gasteiger charges, merging of nonpolar hydrogen atoms, identification and assignment of rotatable bonds, and conversion into PDBQT format suitable for docking analysis.

Molecular docking simulations were performed using AutoDock 4.2 employing the Lamarckian genetic algorithm (LGA). Docking parameters included 100 independent runs, a population size of 150 individuals, a maximum of 2.5 × 10^6^ energy evaluations and 27,000 generations to ensure thorough conformational sampling. The grid box was centred on the enzyme active site and dimensioned to fully encompass the binding pocket and surrounding residues. The resulting docked conformations were clustered based on a root‐mean‐square deviation (RMSD) tolerance of 2.0 Å. The most favourable binding poses were selected according to the lowest predicted binding free energy in conjunction with key intermolecular interactions. Postdocking interaction analysis and visualization of ligand–receptor complexes were performed using Discovery Studio Visualizer v24.1.0.23298.

### 2.7. Catalytic Activity of CSH‐NiONPs

The catalytic performance of the biosynthesized CSH‐NiONPs was evaluated using 4‐NP as a model organic pollutant. The reduction of 4‐NP was conducted in the presence of sodium borohydride (NaBH_4_) within a quartz UV–vis cuvette under ambient conditions. Briefly, 0.2 mL of 4‐NP solution (1.0 mM) was mixed with 0.2 mL of freshly prepared NaBH_4_ solution (0.5 M), leading to the formation of the nitrophenolate ion, as indicated by an intense yellow coloration. Subsequently, 15 μL of a 0.06% (w/v) aqueous suspension of CSH‐NiONPs was introduced into the reaction mixture to initiate the catalytic process. The reaction progress monitored at predetermined time intervals using UV–vis spectroscopy over the wavelength range of 200–500 nm [38]. The reduction of 4‐NP to 4‐aminophenol was confirmed by a gradual decrease in the characteristic absorption peak at *ʎ*_max_ = 410 nm. Quantitative analysis was performed based on the Beer–Lambert law, which correlates absorbance with concentration. The catalytic reduction percentage (CR%) was calculated using equation (1) by comparing the initial concentration of 4‐NP before catalyst addition (*C*_0_) with its concentration at time *t* (*C*_*t*_) after the introduction of CSH‐NiONPs.

(1) CR%=C0−CtC0×100.

The catalytic performance of CSH‐NiONPs was further optimized by systematically evaluating key experimental parameters, including solution pH, catalyst dosage, initial 4‐NP concentration, NaBH_4_ concentration and the presence of coexisting ions. The effect of pH was examined by adjusting the pH of a 14 ppm 4‐NP solution from 0 to 10 using 0.1 M HCl or 0.1 M NaOH. The influence of initial substrate concentration was assessed using 10, 20 and 30 ppm 4‐NP solutions at pH 5°C and 25°C. Catalyst dosage was varied by altering the volume of CSH‐NiONP suspension (15, 25, 35 and 45 μL), while the effect of the reducing agent was investigated using different volumes of NaBH_4_ (5, 20, 45 and 70 μL). To simulate complex aqueous environments, the impact of common coexisting ions (Na^+^, K^+^ and Ca^2+^) was evaluated using 14 ppm 4‐NP solutions. In all experiments, the extent of 4‐NP reduction was monitored using UV–vis spectrophotometry.

#### 2.7.1. Reusability of CSH‐NiONPs

Equimolar volumes (50 mL each) of the as‐synthesized CSH‐NiONPs and NaBH_4_ were added to 100 mL of an aqueous 4‐NP dye solution to initiate the catalytic reduction. The reaction was conducted at room temperature under dark conditions and allowed to proceed until the solution became colourless, indicating complete reduction. After completion, the CSH‐NiONPs were recovered by centrifugation, thoroughly washed with deionized water and methanol to remove residual reactants and dried at 80°C. A 0.06% suspension of the dried CSH‐NiONPs was then used for the first catalytic cycle (Cycle 1) in a UV–vis cuvette. The recovered NPs were subsequently reused in successive cycles under identical dark conditions, with the fourth cycle (Cycle 4) evaluated to assess the catalyst’s reusability and stability.

### 2.8. Statistical Analysis

All experiments were performed in triplicate (*n* = 3), and the results are expressed as mean ± standard deviation (SD). Zones of inhibition (mm) were compared among CSH‐NiONPs, CSH extract and gentamycin for each bacterial strain separately.

## 3. Results and Discussion

### 3.1. Biosynthesis and Characterization of CSH‐NiONPs

**FIGURE 2:**
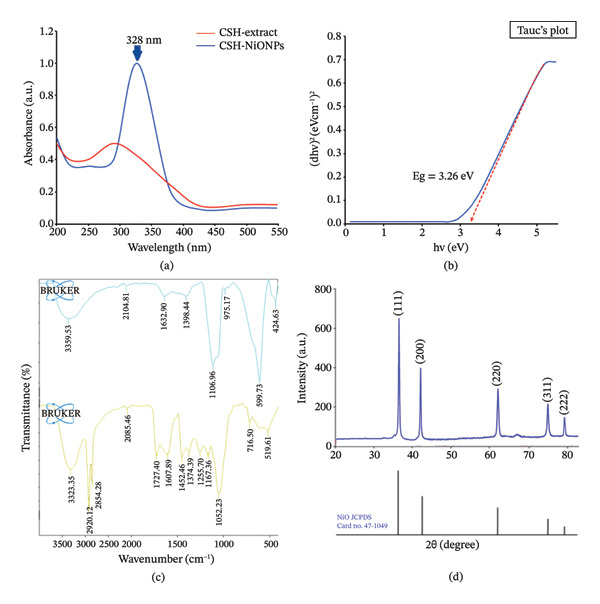
(a) UV–vis spectra of the CSH extract and CSH‐NiONPs, (b) Tauc plot represents the energy bandgap of CSH‐NiONPs, (c) FT‐IR spectra of the CSH extract and CSH‐NiONPs between 500 and 4000 cm^−1^ and (d) XRD pattern of CSH‐NiONPs.

The artichoke head constitutes a rich natural reservoir of bioactive phytochemicals with strong potential for green nanomaterial synthesis. However, plant‐mediated NP fabrication often varies with the composition and concentration of these phytoconstituents. In the present study, CSH‐NiONPs were successfully synthesized within 4 h, as indicated by the colour change from brown to black. The artichoke head extract functioned simultaneously as a reducing and stabilizing (capping) agent, facilitating NP formation and improving colloidal stability. Even at low extract concentrations, the bioactive compounds effectively promoted NP formation while preventing aggregation, yielding stable and well‐dispersed NiO NPs [39]. The optical properties of the colloidal NP dispersion were governed by particle size and morphology via surface plasmon resonance (SPR) and were analysed by UV–vis spectroscopy in the 200–600 nm range to identify the characteristic SPR band. As shown in Figure 2(a), the synthesized CSH‐NiONPs exhibited a distinct absorption maximum at 328 nm, characteristic of nanoscale NiO, thereby confirming successful NP formation [40]. This absorption band was predominantly attributed to ligand‐to‐metal charge‐transfer transitions within the NiO lattice, specifically involving O^2−^ ⟶ Ni^2+^ electronic transitions. The relatively high absorption intensity indicates pronounced quantum confinement effects associated with the nanoscale dimensions of the particles, reflecting reduced particle size and an increased surface‐to‐volume ratio [41]. The optical bandgap energy (Eg) for absorption peak was determined using Tauc’s equation (*α*hν)^1/2^ = *P* (hν − *E*_*g*_), where *α* denotes the absorption coefficient, hν represents the incident photon energy, *P* is a proportionality constant, and *n* defines the nature of the electronic transition (direct or indirect). The estimated bandgap energy was 3.26 eV, obtained by extrapolating the linear portion of the Tauc plot to the energy axis (Figure 2(b)).

FT‐IR spectroscopy was employed to elucidate the functional groups and phytochemical constituents involved in the reduction, capping and stabilization of CSH‐NiONPs (Figure 2(c)). The FT‐IR spectrum of the crude CSH extract exhibited a broad absorption band centred at 3323 cm^−1^, attributed to O–H stretching vibrations of hydroxyl and phenolic groups, indicative of polyphenolic compounds [42]. The band observed at 2920 cm^−1^ corresponds to aliphatic C–H stretching vibrations [42]. A prominent peak at 1607 cm^−1^ was assigned to C=C stretching vibrations of aromatic rings, while the band at 1452 cm^−1^ was associated with C=O stretching vibrations of carbonates [43]. Furthermore, the absorption bands at 1167 and 1052 cm^−1^ were ascribed to C–O and C–O–C stretching vibrations of aromatic acids and ester functionalities, respectively [43]. The peak at 716 cm^−1^ corresponds to H–C–H rocking vibrations in aliphatic compounds, whereas the band around 519 cm^−1^ attributed to metal–oxygen bending vibrations [44]. However, in the FT‐IR spectrum of biosynthesized CSH‐NiONPs, notable shifts and variations in the band intensity were observed, indicating a strong interaction between phytochemicals and the NP surface. The prominent absorption bands at 3359 and 2104 cm^−1^ were attributed to O–H stretching vibrations of hydroxyl and C=C conjugated stretching vibrations of phenolic groups [45], suggesting the involvement of the hydroxyl group in NP capping and stabilization. The band at 1632 cm^−1^ corresponds to C=O stretching vibrations of amide group, while the peak at 1398 cm^−1^ was assigned to symmetric stretching of the carboxylate (COO¯) group and C–H bending vibrations [46]. The sharp band at 1106 cm^−1^ further confirms C–O stretching in esters/saccharides, and the band at 1012 cm^−1^ was associated with C–H deformation modes [47]. The peak observed at 975 cm^−1^ was associated with surface species, such as O–H bending vibrations, residual precursor bending modes or surface‐adsorbed water. Furthermore, the characteristic band at 424 cm^−1^ corresponds to Ni–O lattice vibrations, confirming the formation of NiO NPs [48]. The presence of well‐defined bands within the 400–700‐cm^−1^ region provides strong evidence for Ni–O bond formation, while the absence of additional impurity peaks indicates successful synthesis using the green method.

The crystalline nature and purity of fabricated biogenic CSH‐NiONPs were confirmed by XRD analysis (Figure 2(d)). The XRD pattern displayed five prominent, sharp and well‐defined diffraction peaks at 2*θ* values of 37.41°, 43.23°, 63.21°, 75.48° and 79.32°, which were indexed to the (111), (200), (220), (311) and (222) crystallographic planes, respectively, characteristic of a face‐centred‐cubic (FCC) NiO structure [49]. The absence of any extraneous diffraction peaks indicates the high phase purity of the synthesized NPs. The diffraction profile closely matched the standard reference data from the Joint Committee on Powder Diffraction Standards (JCPDS Card No. 47‐1049) [50]. The prominence of the (111) reflection suggests preferential crystal growth along this orientation. Using the Debye–Scherrer equation, the average crystallite size calculated from the most intense peak was approximately 26.57 nm.

**FIGURE 3:**
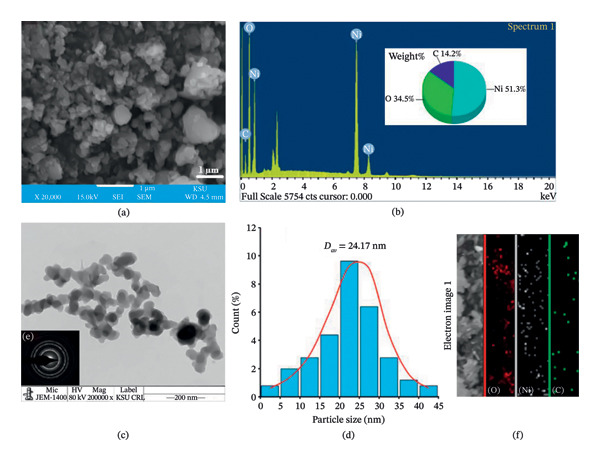
(a) SEM image at 20,000X magnification, (b) EDX analysis, (c) TEM image demonstrating morphology, (d) particle size distribution, (e) SAED diffraction pattern, and (f) elemental mapping of green‐synthesized CSH‐NiONPs.

The surface morphology, aggregation behaviour and elemental composition of the synthesized CSH‐NiONPs were examined using SEM coupled with EDX analysis. SEM micrographs revealed that the synthesized NPs were small, predominantly spherical, morphologically uniform and well‐dispersed, with minimal agglomeration (Figure 3(a)). The particle size estimated from SEM ranged from 18 to 45 nm, consistent with the crystallite size calculated from XRD measurements. EDX spectroscopy confirmed the presence of NiO, showing nickel (Ni, 51.3 wt%) and oxygen (O, 34.5 wt%) as the main components, along with minor contribution from carbon (C, 14.2 wt%) (Figure 3(b)). The strong Ni and O signals corroborate the crystalline phases observed in XRD [51], while the characteristic energy signals within the 0–10 keV range further validate successful NP synthesis [52]. The detected carbon likely originates from the conductive tape used during SEM preparation and residual organic compounds [53]. HR‐TEM analysis supported the spherical morphology of the CSH‐NiONPs (Figure 3(c)), with a particle size distribution of approximately 18.23–45.20 nm and an average size of 24.17 nm, as determined from the TEM histogram using ImageJ software (Figure 3(d)).

SAED analysis verified the crystalline nature of the NPs, exhibiting distinct concentric diffraction rings corresponding to the (111), (200), (220), (311) and (222) planes of the FCC NiO lattice (Figure 3(e)). These results align with the XRD findings, verifying the crystalline, spherical and nanoscale characteristics of the biosynthesized CSH‐NiONPs. In addition, high‐resolution SAED patterns obtained at different magnifications demonstrated uniform crystallinity through the presence of sharp and well‐defined diffraction features [54]. Furthermore, elemental mapping (Figure 3(f)) illustrated uniform distribution of nickel (Ni) and oxygen (O) throughout the nanostructures, confirming the successful formation of the NiO phase. The spatial overlap of Ni and O signals indicates chemical homogeneity across the scanned region. A secondary carbon (C) signal detected was due to the conductive carbon tape used during sample preparation and biocomponents of the plant extract, which were used as a reducing agent. The absence of extraneous peaks verified the high purity of the CSH‐NiONPs.

### 3.2. Antibacterial Activity

**FIGURE 4:**
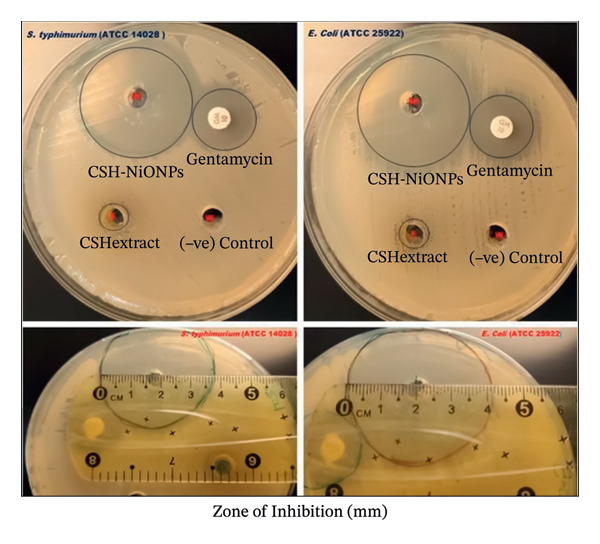
Antibacterial activity of the CSH extract and biosynthesized CSH‐NiONPs against *S. typhimurium* and *E. coli*. The inhibition zone diameters indicate enhanced antibacterial performance in the presence of CSH‐NiONPs compared to the CSH extract. Gentamicin was used as a positive control.

The antibacterial efficacy of the crude CSH extract, CSH‐mediated CSH‐NiONPs and the reference antibiotic gentamycin was systematically assessed against two Gram‐negative bacterial strains, *S. typhimurium* and *E. coli*. Among the evaluated samples, CSH‐NiONPs demonstrated the highest antibacterial efficacy. At a concentration of 10 μg/mL, CSH‐NiONPs produced a remarkably large zone of inhibition against *E. coli* (41.12 ± 0.22 mm), indicating potent bactericidal activity. This enhanced performance can be attributed to the NP‐induced disruption of bacterial membrane integrity and interference with critical cellular processes potentially mediated by strong NP–cell interactions, ROS generation and controlled metal ion release. In contrast, the crude CSH extract exhibited a significantly lower antibacterial activity, producing a zone of inhibition of 10.42 ± 0.12 mm against *E. coli* even at a higher concentration (100 μg/mL). This comparatively reduced efficacy underscores the importance of nanoscale formulation, as the absence of NP‐associated mechanisms limits antimicrobial performance. The superior antibacterial effect of CSH‐NiONPs is due to the synergistic effect between NiONPs and CSH‐derived phytochemicals, which may enhance bacterial membrane permeability and promote intracellular damage. However, the reference antibiotic gentamicin (10 μg/mL) exhibited a moderate inhibition zone (18.25 ± 0.10 mm), which was considerably smaller than that of CSH‐NiONPs, underscoring the enhanced antibacterial potency of the synthesized nanomaterial (Figure 4(a); Table 1). A similar trend was observed against *S. typhimurium*, where CSH‐NiONPs (10 μg/mL) achieved the largest inhibition zone (38.12 ± 0.28 mm). The crude CSH extract (100 μg/mL) showed moderate activity (10.00 ± 0.52 mm), likely due to selective interactions of bioactive compounds with bacterial cellular targets. Gentamycin (10 μg/mL) produced an inhibition zone of 18.16 ± 0.02 mm, consistent with its well‐established broad‐spectrum antibacterial properties (Table 1; Figure 4(b)).

**TABLE 1:** Zone of inhibition of biosynthesized CSH‐NiONPs and CSH extract against selected bacteria.

S. no	Sample	Concentration (μg/mL)	Zone of inhibition (mm)	
			*S. typhimurium* (ATCC 14028)	*E. coli* (ATCC 25922)
1	CSH‐NiONPs	10	38.12 ± 0.28	41.12 ± 0.22
2	CSH extract	100	10 ± 0.52	10.42 ± 0.12
3	Gentamycin	10	18.16 ± 0.02	18.25 ± 0.10

**FIGURE 5:**
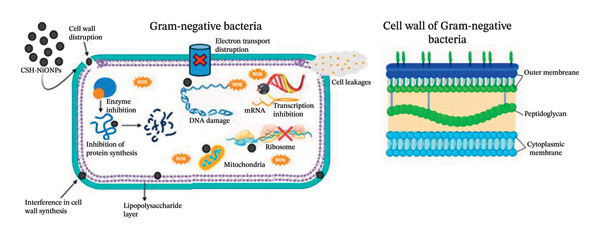
The possible mechanisms for the antibacterial activity of biosynthesized CSH‐NiONPs against *S. typhimurium* and *E. coli*.

Quantitative antibacterial evaluation further substantiated the pronounced antibacterial efficacy of CSH‐NiONPs. The MIC and MBC against *S. typhimurium* were determined to be 3.12 μg/mL and 6.25 μg/mL, respectively, indicating potent growth inhibition and bactericidal activity at notably low concentrations. In comparison, MIC and MBC values against *E. coli* were 9.76 μg/mL and 19.53 μg/mL, respectively. These findings demonstrate that although CSH‐NiONPs exhibit a substantial antibacterial activity against both Gram‐negative strains, *S. typhimurium* displays comparatively higher susceptibility (Table 2). The observed antibacterial performance is strongly influenced by NP size, with smaller particles providing an increased surface‐to‐volume ratio that facilitates enhanced interaction with bacterial cells and promotes ROS generation. These ROS readily interact with Gram‐negative bacteria, which are structurally characterized by a relatively thin peptidoglycan layer and an outer membrane, making them more susceptible than Gram‐positive counterparts possessing a thicker peptidoglycan barrier [55]. The bactericidal activity of CSH‐NiONPs is mediated through multiple synergistic mechanisms, including the disruption of the cell wall and cytoplasmic membrane, induction of membrane deformation and permeability loss leading to cytoplasmic leakage, as well as intracellular damage such as DNA fragmentation and protein denaturation. Additionally, ROS species—including hydrogen peroxide (H_2_O_2_), hydroxyl radicals (•OH) and superoxide anions (O_2_•^−^)—intensify oxidative stress, ultimately resulting in cellular dysfunction and death [56]. Moreover, the positively charged surface of CSH‐NiONPs enhances electrostatic interactions with negatively charged bacterial membranes, promoting membrane destabilization, leakage of intracellular constituents and subsequent biomolecular degradation (Figure 5).

**TABLE 2:** MIC and MBC of CSH‐NiONPs against selected bacteria.

Tested microorganisms	CSH‐NiONPs	
	MIC (μg/mL)	MBC (μg/mL)
*S. typhimurium* (ATCC 14028)	3.12	6.25
*E. coli* (ATCC 25922)	9.76	19.53

These findings demonstrate the exceptional antibacterial efficacy of CSH‐NiONPs against the prominent waterborne pathogens *S. typhimurium* and *E. coli*. The markedly large zones of inhibition (≈38–41 mm) in conjunction with notably low MIC and MBC values highlight their strong bactericidal potency at minimal dosages. This pronounced antibacterial activity can be attributed to the synergistic effects of nanoscale size, enhanced surface reactivity and bioactive phytochemical capping agents derived from the plant extract, which facilitate effective bacterial membrane disruption and intracellular damage. Such superior antimicrobial performance highlights the substantial potential of plant‐mediated CSH‐NiONPs for rapid and efficient bacterial eradication, particularly in water decontamination applications under emergency or high‐risk contamination conditions. Their incorporation into advanced water treatment systems could significantly improve microbial safety by achieving efficient pathogen removal at reduced concentrations, thereby minimizing reliance on conventional chemical disinfectants. Furthermore, the integration of CSH‐NiONPs into filtration matrices or disinfection units represents a promising and sustainable approach for enhancing drinking water quality with potentially lower environmental impact [57]. From a public health perspective, the deployment of these nanomaterials could contribute meaningfully to outbreak mitigation strategies, especially in resource‐limited settings where access to safe water and sanitation infrastructure remains a critical challenge [58]. The antibacterial activity of the biosynthesized CSH‐NiONPs was compared with previously reported green‐synthesized NiONPs and demonstrated superior performance, as summarized in Table 3.

**TABLE 3:** Comparison of the antibacterial activity of biosynthesized CSH‐NiONPs with previously reported green‐synthesized NiONPs.

Nanoparticle	Plant used	Bacterial strains	Nanoparticle concentration	Inhibition zone	Reference
NiONPs	*M. oleifera* leaves	*E. coli*, *S. aureus* (mutans)	0.0315 μg/mL	13–14 mm	[59]
NiONPs	*A. cepa* stalks	*S. aureus*	0.025 mg/mL	11 mm	[60]
NiONPs	*A. marmelos* leaves	*E. coli*, *S. aureus*, *S. mutans*, *B. subtilis*	25 μg/mL	14–18 mm	[30]
NiONPs	*B. tomentosa* seed pods and leaves	*E. coli*, *P. aeruginosa*, *S. aureus*	500 μg/mL	11.70–20.40 mm	[61]
NiONPs	*P. tomentosa* leaves	*E. coli*, *P. aeruginosa*, *A. baumannii*, *K. pneumoniae*, *E. aerogenes*, *S. dysentery*, *S. aureus*, *B. cereus*, *E. faecalis*, *S. epidermidis*, *L. monocytogenes*.	50 μg/mL	21–30 mm	[62]
NiONPs	*S. persica* roots	*E. coli*, *E. cloacae*, *K. pneumoniae*, *P. aeruginosa*, *S. epidermidis*, *E. faecalis*, *S. aureus*, *S. pneumoniae*	100 μL	18–31 mm	[63]
NiONPs	*C. roseus* leaves	*E. coli*, *P. aeruginosa*, *B. subtilis*	60 μg/mL	15–22 mm	[64]
NiONPs	*A. nilotica* leaves	*E. coli*, *P. aeruginosa*, *S. aureus*, *B. subtilis*	100 μg/mL	14–18 mm	[65]
NiONPs	*A. vera* gel	*E. coli*, *P. multocida*, *B. subtilis*, *S. aureus*	50 μg/mL	17–25 mm	[50]
NiONPs	*P. ciliata* leaves	*E. coli*, *K. pneumoniae*, *B. subtilis*, *B. licheniformis*	8 mg/mL	15.9–28.1 mm	[66]
NiONPs	*S. rebaudiana* leaves	*E. coli*, *B. subtilis*, *S. pneumonia*	0.1 mg/mL	12–16 mm	[67]
NiONPs	Okra leaves	*E. coli*, *P. aeruginosa*, *S. aureus*	150 μg/mL	10 mm	[68]
NiONPs	*O. sanctum* leaf extract	*E. coli*, *K. pneumoniae*, *S. typhi*, *B. subtilis*, *S. epidermidis*	100 μg/mL	18.6–25.1 mm	[69]
CSH‐ NiONPs	*C. scolymus* head (globe)	*E. coli*, *S. typhimurium*	10 μg/mL	18.25–41.12 mm	This work

### 3.3. Molecular Docking

**FIGURE 6:**
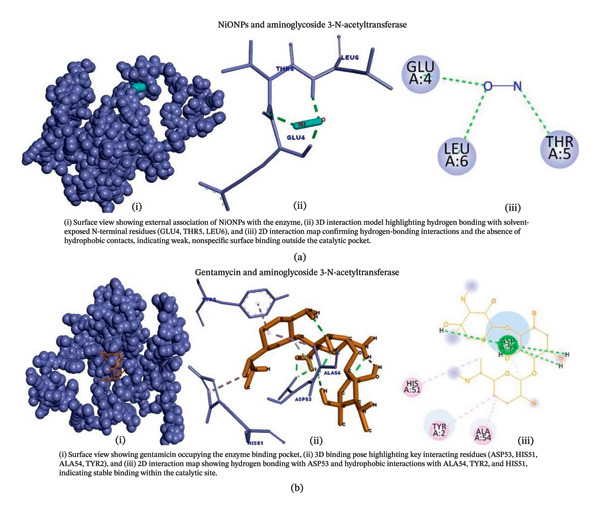
(a) Docking‐based analysis of the interaction between nickel oxide nanoparticles (NiONPs) and aminoglycoside 3‐N‐acetyltransferase and (b) representative docking poses of gentamicin with aminoglycoside 3‐N‐acetyltransferase.

Molecular docking simulations were performed to comparatively assess the interaction profiles of biosynthesized CSH‐NiONPs and gentamicin with AAC(3), a key resistance enzyme that inactivates aminoglycoside through acetylation. The primary objective was to elucidate whether CSH‐NiONPs could bind to or sterically hinder the enzyme’s catalytic domain, thereby modulating its activity. The docking results revealed that NiONPs exhibit weak and nonspecific interactions with AAC(3), as reflected by a low binding affinity (−1.86 kcal·mol^−1^) and a single, highly convergent docking pose (cluster RMSD = 0.00 Å), indicative of limited conformational variability but poor thermodynamic favourability. Although 12 hydrogen bonds were detected, these interactions were predominantly localized to solvent‐accessible N‐terminal residues (GLU4, THR5 and LEU6) and lacked complementary hydrophobic or π‐mediated stabilization. The spatial restriction of these contacts to peripheral, noncatalytic regions—coupled with the absence of interactions within the active or substrate‐binding pocket—strongly suggests that NiO NPs do not effectively engage functionally critical residues of AAC (3). This interaction pattern is characteristic of nonspecific surface association rather than biologically relevant binding and thus is unlikely to contribute to enzymatic the recognition or acetylation of aminoglycoside substrates (Table 4; Figure 6(a)).

**TABLE 4:** Comparative molecular docking analysis of aminoglycoside 3‐N‐acetyltransferase with NiO and gentamicin.

Protein (receptor) and ligand	Binding energy (kcal/mol)	Cluster RMSD (A)	No. of H bonds	Amino acids involved in H‐bond interaction	Hydrophobic interactions	Amino acids involved in hydrophobic interactions
Aminoglycoside 3‐N‐acetyltransferase and NiO	−1.86	0.00	12	GLU4; THR5; LEU6	Nil	Nil
Aminoglycoside 3‐N‐acetyltransferase and gentamycin	−7.82	0.00	7	ASP53	3	ALA54; TYR2; HIS51

*Note:* This table summarizes the docking interactions of aminoglycoside 3‐N‐acetyltransferase with nickel oxide (NiO) and gentamicin. NiO shows a weak, low‐affinity surface binding (−1.86 kcal/mol; RMSD = 0.00 Å), forming hydrogen bonds only with solvent‐exposed N‐terminal residues (GLU4, THR5, LEU6) and no hydrophobic contacts, indicating nonspecific, noncatalytic association. In contrast, gentamicin exhibits a strong and specific binding (−7.82 kcal/mol; RMSD = 0.00 Å), engaging ASP53 via hydrogen bonding and ALA54, TYR2 and HIS51 through hydrophobic interactions, consistent with stable active‐site accommodation.

In contrast, gentamicin demonstrated strong and specific interaction with AAC(3), characterized by a significantly more favourable binding energy (−7.82 kcal·mol^−1^) and a stable docking conformation (cluster RMSD = 0.00 Å). The ligand established seven hydrogen bonds, predominantly involving the catalytically critical residue ASP53, along with three hydrophobic interactions with ALA54, TYR2 and HIS51 (Table 4; Figure 6(b)).

These interactions facilitate stable accommodation within a well‐defined binding pocket and are consistent with productive substrate recognition. This binding mode aligns with the established biochemical function of AAC (3) in mediating gentamicin acetylation and subsequent antibiotic inactivation, thereby conferring resistance [70, 71]. The pronounced contrast between the two ligands underscores that gentamicin is the biologically competent substrate that effectively engages the catalytic site of AAC (3), whereas NiO lacks the structural complementarity and energetic favourability required for active‐site binding. Notably, NiO neither stabilizes the enzyme–substrate complex nor competes for occupancy within the catalytic pocket, indicating that it does not potentiate aminoglycoside resistance through the direct modulation of AAC (3) activity. Instead, its weak and surface‐localized interaction profile suggests an inability to participate in or facilitate enzymatic catalysis. Thus, these findings demonstrate that NiO does not function as a molecular effector of aminoglycoside resistance at the enzymatic level. The absence of meaningful binding or stabilization of AAC (3) supports the potential application of CSH‐NiONPs as antibacterial agents with a mechanism of action that is independent of aminoglycoside resistance pathways. Such mechanistic decoupling is advantageous for antimicrobial design, as it enables effective bacterial inhibition while minimizing the likelihood of inducing or exacerbating enzyme‐mediated resistance [72, 73].

### 3.4. Catalytic Analysis of CSH‐NiONPs

**FIGURE 7:**
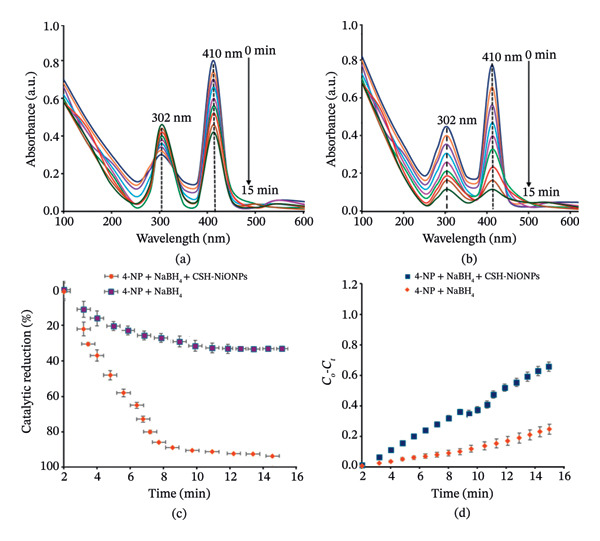
UV–vis absorption spectra of 4‐NP during a 15‐min catalytic reduction: (a) in the absence of CSH‐NiONPs, (b) in the presence of CSH‐NiONPs, (c) calculated CR% efficiency of 4‐NP, and (d) zero‐order kinetic plot for 4‐NP reduction (pH = 5, *C*_0_ = 14 ppm, *T* = 278 K).

The catalytic performance of the synthesized CSH‐NiONPs was systematically investigated for the reduction of waterborne contaminants, using 4‐NP as a model pollutant. The biosynthesized CSH‐NiONPs demonstrated pronounced heterogeneous catalytic activity, underscoring their potential for the effective remediation of persistent and toxic organic species in water. The reduction reaction was conducted in the presence of NaBH_4_ as a reducing agent, with CSH‐NiONPs serving as the catalyst. The reaction kinetics and progression were monitored by time‐dependent UV–vis absorption spectroscopy. As illustrated in Figures 7(a,b), the UV–vis spectra of 4‐NP recorded in the absence and presence of CSH‐NiONPs, respectively, revealed distinct spectral changes. Pristine 4‐NP displayed two characteristic absorption bands at approximately 306 and 410 nm. Upon the addition of NaBH_4_ to the aqueous 4‐NP solution, a single intense absorption peak at ∼410 nm appeared, corresponding to the formation of 4‐nitrophenolate ions [74].

In the absence of a catalyst, the absorbance at 410 nm remained nearly unchanged over time, indicating a negligible reduction of 4‐NP under identical conditions. Conversely, the introduction of CSH‐NiONPs into the NaBH_4_–4‐NP system resulted in a rapid decline in the absorbance intensity at 410 nm, accompanied by the progressive emergence of a new absorption band at ∼306 nm, corresponding to the formation of 4‐aminophenol (4‐AP). The reaction kinetics, monitored over a time interval of 0–15 min (Figure 7(b)), revealed a significant attenuation of the 4‐nitrophenolate peak alongside a concurrent increase in the 4‐AP signal within the initial stages of the reaction, confirming the high catalytic efficiency of the nanomaterial. Notably, a complete reduction of 4‐NP to 4‐AP was achieved within 15 min in the presence of CSH‐NiONPs, whereas the reaction proceeded insignificantly without the catalyst. For quantitative assessment, the catalytic reduction efficiency (CR%) was determined using equation (1), with the corresponding results presented in Figure 7(c). Approximately 95% conversion of 4‐NP was achieved within 15 min using CSH‐NiONPs, compared to only 39% reduction in the catalyst‐free system over the same duration. The enhanced catalytic performance can be attributed to the nanoscale dimensions and high surface‐to‐volume ratio of CSH‐NiONPs, which provide abundant active sites and facilitate efficient electron transfer between BH_4_^-^ ions and 4‐NP molecules, consistent with previously reported findings [75, 76]. To further elucidate the reaction kinetics, the experimental data were analysed using a zero‐order kinetic model, expressed in its linearized form as

(2) C0−Ct=kt,

where *k* is the apparent rate constant (mol·L^−1^·min^−1^), *t* is the reaction time (min), and *C*_0_ and *C*_*t*_ represent the initial and final concentrations (mol·L^−1^) of 4‐NP before and after catalytic reduction, respectively. As depicted in Figure 7(d), linear regression analysis yielded a high correlation coefficient (*R* ^2^ > 0.978), confirming that the reduction process follows zero‐order kinetics under the applied conditions.

The apparent rate constant for the catalytic reduction of 4‐NP in the presence of CSH‐NiONPs and NaBH_4_ was determined to be 0.048 mol·L^−1^·min^−1^ (Table 5), significantly exceeding that of the catalyst‐free system (0.017 mol·L^−1^·min^−1^). These findings clearly demonstrated the superior catalytic efficiency of the synthesized CSH‐NiONPs relative to previously reported systems [77].

**TABLE 5:** Comparison of kinetic parameters for 4‐NP reduction with and without CSH‐NiONPs.

Catalyst	Regression equation	*k* (mol·L^−1^·min^−1^)	*R* ^2^
4‐NP + NaBH_4_	*Y* = 0.017*x* − 0.0327	0.017	0.9785
4‐NP + NaBH_4_ + CSH‐NiONPs	*Y* = 0.048*x* − 0.0026	0.048	0.9782

The superior catalytic performance of the synthesized CSH‐NiONPs can be attributed to the green synthesis route employing CSH extract, in which bioactive phytoconstituents act concurrently as reducing and stabilizing agents. This biogenic approach promotes the formation of ultrafine, well‐dispersed NiONPs with high surface areas. Such structural and physicochemical characteristics significantly increase the density of accessible active sites and facilitate efficient electron transfer from donor species (BH_4_^−^ ions) to acceptor molecules (4‐NP) at the catalyst interface. As a result, the reduction reaction is substantially accelerated, highlighting the potential of CSH‐NiONPs as efficient heterogeneous catalysts for environmentally sustainable remediation processes.

#### 3.4.1. Effect of Reaction Parameters on the Catalytic Reduction of 4‐NP

**FIGURE 8:**
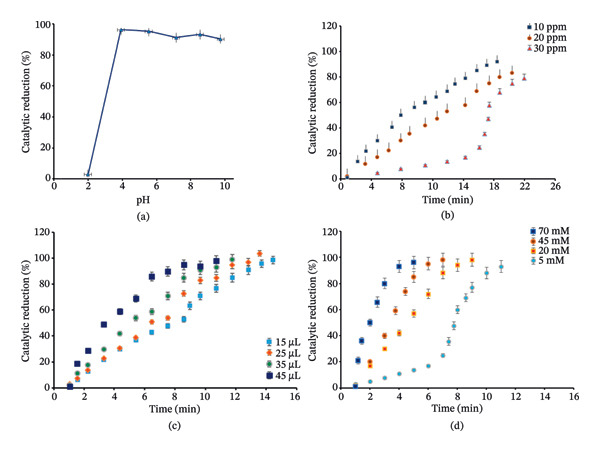
Effect of reaction parameters on the catalytic reduction of 4‐NP: (a) pH (*C*_0_ = 14 ppm, CSH‐NiONPs = 15 μL of 0.06%, NaBH_4_ = 1 mL of 10 mM); (b) initial 4‐NP concentration (pH = 5, CSH‐NiONPs = 15 μL of 0.06%, NaBH_4_ = 1 mL of 10 mM); (c) CSH‐NiONPs dosage (pH = 5, *C*_0_ = 14 ppm, NaBH_4_ = 1 mL of 10 mM); and (d) NaBH_4_ dosage (pH = 5, *C*_0_ = 14 ppm, CSH‐NiONPs = 15 μL of 0.06%).

The influence of key operational parameters, including solution pH, initial 4‐NP concentration, CSH‐NiONP dosage and NaBH_4_ concentration, was systematically investigated to optimize the catalytic reduction conditions. The effect of pH was investigated across a broad range (pH 2, 4, 6, 8 and 10), while other experimental conditions such as reactant concentrations, catalyst loading, reaction time and temperature were kept constant. Solution pH plays a critical role in determining both the surface charge characteristics of the catalyst and the ionization state as well as adsorption behaviour of 4‐NP, thereby directly influencing the reduction efficiency. As shown in Figure 8(a), the reduction efficiency increased markedly with increasing pH (2–10), reaching optimal performance under mildly alkaline conditions (pH > 4). This enhancement can be attributed to the development of a negatively charged catalyst surface above its zero point of charge (ZPC), which facilitates favourable electrostatic interactions with protonated 4‐NP species, thereby promoting their adsorption and enhancing interfacial electron transfer, in agreement with previous findings [78]. The effect of initial 4‐NP concentration was further examined using aqueous solutions of 10, 20 and 30 ppm, while maintaining constant pH, catalyst dosage, NaBH_4_ concentration and temperature. As depicted in Figure 8(b), increasing the 4‐NP concentration led to a proportional increase in the time required for complete reduction, with reaction durations extending to approximately 7, 15 and 22 min for 10, 20 and 30 ppm solutions, respectively. This trend can be ascribed to the higher number of 4‐NP molecules competing for a limited number of active sites on the catalyst surface at elevated concentrations, resulting in reduced catalytic efficiency per unit time [79].

The impact of catalyst dosage on catalytic reduction efficiency was examined using 1 mM 4‐NP and 1 mL of 10 mM NaBH_4_, with varying volumes (15, 25, 35 and 45 μL) of 0.06% CSH‐NiONPs, as illustrated in Figure 8(c). The results indicate that increasing the dosage of the catalyst significantly shortened the reaction time to approximately 15, 10, 5 and 2 min, respectively. This improvement can be attributed to the greater availability of active catalytic sites at higher dosages, which enhances the adsorption of reactant species and accelerates the overall reduction kinetics [80]. Similarly, the effect of the NaBH_4_ concentration on catalytic reduction was evaluated using 1 mM 4‐NP (2 mL), 15 μL of 0.06% CSH‐NiONPs and varying NaBH_4_ concentrations (5, 20, 45 and 70 mM), as shown in Figure 8(d). The results reveal that increasing the NaBH_4_ concentration significantly reduced the time required to achieve approximately 95% catalytic reduction, decreasing from 15 to 9, 5 and 2 min, respectively. This trend reflects the enhanced availability of hydride ions (BH_4_^−^), which facilitates faster electron transfer to 4‐NP molecules via the catalyst surface, thereby accelerating the reduction process [79].

#### 3.4.2. Evaluation of Catalyst Stability and Reusability

**FIGURE 9:**
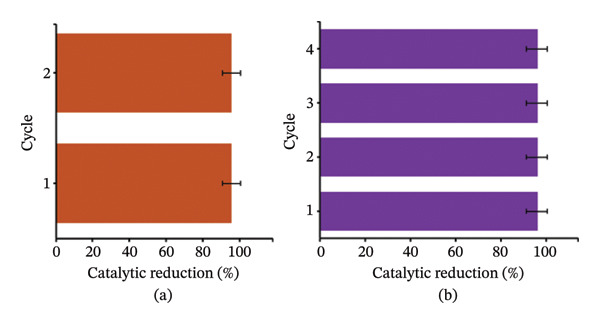
(a) Stability analysis and (b) catalytic reusability performance of biogenic CSH‐NiONPs.

The influence of coexisting ions on the catalytic performance was evaluated in the presence of common aqueous ions, namely, Na^+^, K^+^ and Ca^2+^, and the results are presented in Figure 9(a). The reduction of 4‐NP in the first cycle exhibited behaviour comparable to that observed in the second cycle conducted with these interfering ions, indicating that K^+^, Na^+^ and Ca^2+^ exert negligible effects on the catalytic activity of CSH‐NiONPs. These results underscore the high selectivity of CSH‐NiONPs toward 4‐NP reduction. The reusability of the catalyst was further evaluated to assess its stability and durability during the NaBH_4_‐assisted reduction. As shown in Figure 9(b), CSH‐NiONPs retained high catalytic efficiency over four successive cycles, demonstrating excellent recyclability. After each cycle, the catalyst was recovered by centrifugation, thoroughly washed with deionized water and dried at 100°C for 2 h prior to reuse. Although a slight decline in reduction efficiency was observed upon repeated use, the catalyst maintained substantial activity, likely due to the sustained synergistic interaction between CSH‐NiONPs and NaBH_4_.

#### 3.4.3. Catalytic Reduction Mechanism of CSH‐NiONPs

**FIGURE 10:**
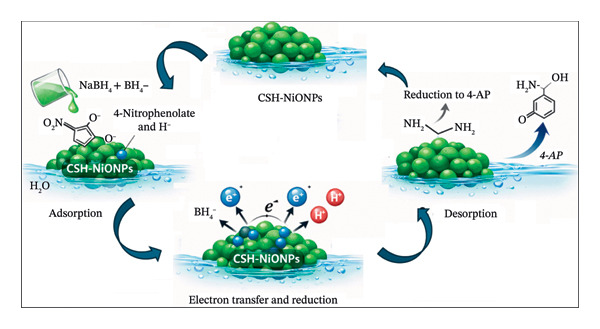
Schematic diagram illustrating the reduction mechanism of 4‐nitrophenol (4‐NP), in which CSH‐NiONPs act as an electron relay to facilitate electron transfer from BH_4_^-^ to the nitro group, resulting in its conversion to 4‐aminophenol (4‐AP) and achieving a complete reduction within 15 min at the optimal catalyst loading.

The catalytic reduction process is initiated by the adsorption of both NaBH_4_ and 4‐NP onto the surface of CSH‐NiONPs through electrostatic interactions and coordination with surface‐bound functional groups derived from phytochemicals in the CSH extract. The conversion of 4‐NP to 4‐aminophenol (4‐AP) in aqueous media proceeds through a three‐step mechanism: (i) adsorption of 4‐nitrophenolate ions and hydride species onto the catalyst surface, (ii) surface‐mediated electron transfer from BH_4_^−^ ions to the adsorbed 4‐nitrophenolate species, leading to the reduction of the nitro group to an amino group, and (iii) desorption of the formed 4‐AP molecules from the catalyst surface [81]. Upon the addition of NaBH_4_ to the aqueous 4‐NP solution containing CSH‐NiONPs, rapid adsorption of 4‐nitrophenolate ions occurs, followed by efficient electron relay from BH_4_^-^ ions to the adsorbed substrate, with CSH‐NiONPs acting as an effective electron‐transfer mediator. This process facilitates the rapid formation of 4‐AP in the presence of available protons in the reaction medium. The green‐synthesized CSH‐NiONPs demonstrated remarkable catalytic efficiency, achieving near‐complete conversion within 15 min and outperforming many previously reported catalytic systems. This enhanced catalytic activity could be primarily attributed to the surface functionalization and stabilization conferred by phytochemicals in the CSH extract, which improve substrate adsorption, prevent NP agglomeration and facilitate effective electron transfer. Figure 10 illustrates the catalytic mechanism using CSH‐NiONPs and NaBH4 against 4‐NP. Compared to conventional NiO‐based NP synthesis, which is often time‐consuming, costly and unstable over repeated cycles, the CSH‐mediated approach offers a rapid, economical and eco‐friendly route, producing CSH‐NiONPs with excellent catalytic efficiency and notable antibacterial activity, underscoring their potential for water remediation applications. As summarized in Table 6, the biogenic CSH‐NiONPs exhibited an enhanced catalytic activity relative to previously reported biosynthesized NiONPs.

**TABLE 6:** Comparison of the catalytic activity of biosynthesized CSH‐NiONPs with previously reported green‐synthesized NiONPs.

Catalyst	Plant used	Dye concentration	Catalyst dose	Degradation (%)	Reference
NiONPs	*P. granatum* leaf extract	3 ppm MB and CR	30 mg/50 mL	91.7, 81.2	[48]
NiONPs	*L. albus* seeds	50 ppm MB	50 mg/15 mL	97	[80]
NiONPs	*P. granatum* peel extract	0.25 mg/L MB	50 mg/25 mL	86	[82]
NiONPs	*P. dodecandra* leaves	10 ppm	20 mg/mL	78.3	[83]
NiONPs	Arabic gum	20 mg/L MB	10 mg/L	82	[84]
NiONPs	*P. granatum* juice extract	10 mg/L MB	0.1 g/50 mL	90	[85]
NiONPs	*D. maritima* roots	Aromatic content	10 mg/50 mL	50–60	[86]
NiONPs	Okra leaves	20 mg/500 mL	3 mg/50 mL	78	[68]
CSH‐NiONPs	*C. scolymus* head	14 ppm 4‐NP	0.6 g/mL	95	This work

*Note:* CR, Congo red; MB, methylene blue; and 4‐nitrophenol.

## 4. Conclusion

This study presents a simple, cost‐effective and eco‐friendly bio‐reduction strategy for synthesizing CSH‐mediated NiONPs, utilizing phytochemicals in the CSH extract as natural reducing and stabilizing agents. Comprehensive characterization (UV–vis, FT‐IR, XRD, SEM, EDX, TEM) confirmed the formation of phytochemically capped NiONPs with a negative surface charge and particle sizes of 18–45 nm. The biosynthesized CSH‐NiONPs demonstrated a strong antibacterial activity against *E. coli* (41.12 ± 0.22 mm) and *S. typhimurium* (38.12 ± 0.28 mm), surpassing gentamicin and plant extracts at lower concentrations, as evidenced by significant inhibition zones and low MIC/MBC values. Molecular docking revealed that gentamicin binds strongly and specifically to AAC(3), whereas NiONPs exhibited only weak, nonspecific interactions, suggesting minimal risk of promoting resistance. Additionally, CSH‐NiONPs acted as efficient heterogeneous catalysts for NaBH_4_‐assisted 4‐NP reduction, achieving ∼95% conversion within 15 min under zero‐order kinetics (*k* = 0.048 mol·L^−1^·min^−1^, *R* ^2^ > 0.97). Thus, the integrated experimental and computational results establish CSH‐NiONPs as multifunctional and sustainable nanomaterials for water purification, exhibiting rapid antimicrobial activity, a minimal propensity to promote resistance mechanisms and efficient catalytic degradation of organic pollutants, thereby highlighting their strong potential for enhancing microbial safety in water treatment systems.

## Funding

This research project was supported by Ongoing Researchers Funding Program (No. ORF‐2026‐878), King Saud University, Riyadh, Saudi Arabia.

## Consent

The authors have nothing to report.

## Conflicts of Interest

The authors declare no conflicts of interest.

## Data Availability

The data that support the findings of this study are available on request from the corresponding author. The data are not publicly available due to privacy or ethical restrictions.
